# Quantitative Analysis of Focused A-To-I RNA Editing Sites by Ultra-High-Throughput Sequencing in Psychiatric Disorders

**DOI:** 10.1371/journal.pone.0043227

**Published:** 2012-08-17

**Authors:** Hu Zhu, Daniel J. Urban, Jared Blashka, Matthew T. McPheeters, Wesley K. Kroeze, Piotr Mieczkowski, James C. Overholser, George J. Jurjus, Lesa Dieter, Gouri J. Mahajan, Grazyna Rajkowska, Zefeng Wang, Patrick F. Sullivan, Craig A. Stockmeier, Bryan L. Roth

**Affiliations:** 1 Department of Pharmacology, University of North Carolina Chapel Hill Medical School, Chapel Hill, North Carolina, United States of America; 2 Department of Genetics, School of Medicine, Chapel Hill, North Carolina, United States of America; 3 Department of Psychology, Case Western Reserve University, Cleveland, Ohio, United States of America; 4 Department of Psychiatry, Case Western Reserve University, Cleveland, Ohio, United States of America; 5 Department of Psychiatry, Louis Stokes Cleveland VA Medical Center, Cleveland, Ohio, United States of America; 6 Center for Psychiatric Neuroscience, Department of Psychiatry and Human Behavior, University of Mississippi Medical Center, Jackson, Mississippi, United States of America; University of Florida, United States of America

## Abstract

A-to-I RNA editing is a post-transcriptional modification of single nucleotides in RNA by adenosine deamination, which thereby diversifies the gene products encoded in the genome. Thousands of potential RNA editing sites have been identified by recent studies (e.g. see Li et al, *Science* 2009); however, only a handful of these sites have been independently confirmed. Here, we systematically and quantitatively examined 109 putative coding region A-to-I RNA editing sites in three sets of normal human brain samples by ultra-high-throughput sequencing (uHTS). Forty of 109 putative sites, including 25 previously confirmed sites, were validated as truly edited in our brain samples, suggesting an overestimation of A-to-I RNA editing in these putative sites by Li et al (2009). To evaluate RNA editing in human disease, we analyzed 29 of the confirmed sites in subjects with major depressive disorder and schizophrenia using uHTS. In striking contrast to many prior studies, we did not find significant alterations in the frequency of RNA editing at any of the editing sites in samples from these patients, including within the 5HT_2C_ serotonin receptor (*HTR2C*). Our results indicate that uHTS is a fast, quantitative and high-throughput method to assess RNA editing in human physiology and disease and that many prior studies of RNA editing may overestimate both the extent and disease-related variability of RNA editing at the sites we examined in the human brain.

## Introduction

Adenosine-to-inosine (A-to-I) RNA editing is a post-transcriptional modification of RNA transcripts catalyzed by ADARs (***A***denosine ***D***eaminases ***A***cting on ***R***NA). A-to-I RNA editing converts genomically encoded adenosine to inosine, which is recognized as guanosine by the translational apparatus [1], [2]. Until recently, only two dozen edited human genes were documented, with the majority involved in central nervous system functions [3], [4], [5], [6], [7], [8]. The high proportion of brain-derived edited mRNAs is not surprising given the high levels of expression of ADARs in the brain [9], [10]. RNA editing has been shown to be important during the development of the brain [11] and ADAR1 knockout mice are embryonically lethal [12] while ADAR2 knockout mice display progressively severe epilepsy and, ultimately, death [13]. In flies, inactivation of A-to-I RNA editing activity (e.g., in dADAR mutant flies) also yields a neurological phenotype with locomotor deficits, seizures and neurodegeneration [14].

In the mammalian brain, RNA editing finely tunes the functions of many proteins involved in electrical and chemical neurotransmission [3], [7], [13], [15], [16]. The RNA editing of subunit 2 of alpha-amino-3-hydroxy-5-methyl-4-isoxazolepropionic acid (AMPA) glutamate receptor (GluR2), by changing the amino acid from glutamine (Q) to arginine (R), leads to Ca^2+^ impermeability of AMPA receptor containing GluR2 subunit [17]. The unedited form of GluR2 mRNA is expressed in the various regions of the developing brain, however nearly 100% edited form of GluR2 mRNA is expressed in the adult brain [17]. The disturbance of RNA editing of GluR2 by the inactivation of ADAR2 results in the progressively epilepsy and the death of mice in a few weeks [13]. The serotonin receptor HTR2C is the only G protein coupled receptor shown to undergo RNA editing, with five editing sites (A, B, E, C, D) within close proximity in the second intracellular loop of the receptor. RNA editing of HTR2C receptor decreases the efficacy of the interaction between the HTR2C and its G proteins, and thus modulates serotonin signaling [3]. Dysregulated editing of HTR2C results in the constitutive activation of the sympathetic nervous system and increased energy expenditure [18] and a Prader-Willi-like syndrome [19]. In the human potassium voltage-gated channel KCNA1, RNA editing recodes a highly conserved isoleucine to a valine, thereby allowing the edited channels to recover from inactivation about 20 times faster than their unedited counterparts. This change in function of KCNA1 greatly influences the action potential shape, signal propagation and the firing pattern [20]. The alpha3 subunit of GABAA receptors has also been shown to undergo RNA editing, with an isoleucine to a methionine change in the third transmembrane region [7]. Such editing substantially alters GABA sensitivity and the deactivation rate of GABA-A receptors, with the unedited form showing a lower GABA EC_50_ and slower decay [21].

In contrast to these small scale studies, recent years have seen an increasing number of potential A-to-I RNA editing sites identified in the human transcriptome by genome wide analysis [4], [22], [23], [24], [25], [26], [27], [28], [29], [30]. Based on the A-to-G discrepancies between genomic and cDNA sequences from human gene database, Athanasiadis et al [22] and Levanon et al [23] found 14,500 and 12,723 editing sites in the human transcriptome, respectively. To directly identify inosines on RNA strands, Sakurai et al [26] developed a chemical method (Inosine chemical erasing, ICE) and found 5,072 editing sites, including 4,395 new sites in human transcriptome. Using a massively parallel target capture and DNA sequencing approach, Li et al [24] detected several hundred l RNA editing sites by comparing genomic DNA with RNA from seven tissues of one individual. However, of the thousands of potential A-to-I RNA editing sites identified by previous studies, only a handful have been independently validated–typically via non-quantitative and low-throughput Sanger sequencing methods [4], [11], [23], [24], [26]. To accurately and quantitatively verify the frequency of editing at these potential RNA editing sites, we recently developed an ultra-high-throughput sequencing approach [31] suitable for ***quantitatively*** analyzing hundreds of potential RNA editing sites simultaneously in normal and diseased tissues. Here, we used this uHTS approach to examine 109 RNA editing sites in coding regions of human brain transcriptome and analyzed 29 confirmed sites in psychiatric disorders. We found that the extent of RNA editing from the sites we examined in Li’s study [24] may be overestimated in normal human brain, and the scale of RNA editing alterations may also be overstated in psychiatric disorders.

## Results and Disscussion

### Validation of 109 Putative A-to-I RNA Editing Sites from Previous Study in Human Brain

**Figure 1:**
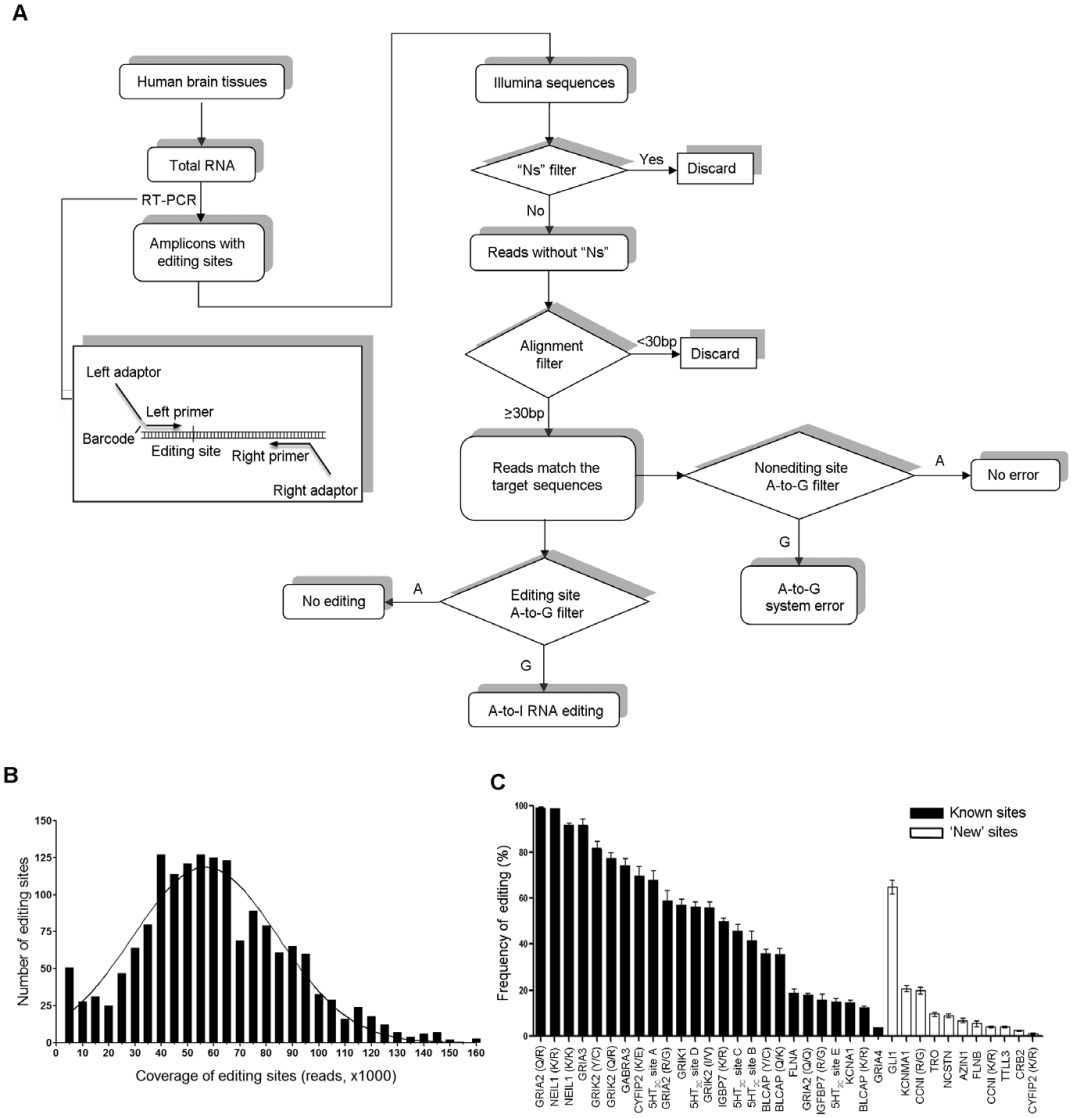
Ultra-high-throughput sequencing of potential A-to-I RNA editing sites. **A**. Shows a schematic diagram of processing and measuring RNA editing using ultra High Throughput Sequencing technology. **B**. Shows the frequency distribution of sequencing coverage for each editing site. The reads for each editing site from three sets of normal human brain samples were grouped in intervals of 5,000 reads. Using the D’Agostino-Pearson normality test, the data do not differ significantly from a Gaussian distribution. **C**. Shows the A-to-I RNA editing frequency of 36 sites from category I, including 11 ‘new’ sites and 25 known sites in three sets of normal human samples. RNA editing frequency is presented as mean, expressed as a percentage of the total population of transcripts, ± SEM.

A-to-I RNA editing primarily occurs in the non-coding regions of RNA, typically in Alu repeats, where it may indirectly affect gene function by altering the spatiotemporal profiles of gene expression [23]. RNA editing events that “re-code” pre-mRNAs in the coding region are particularly important as they can directly alter the biophysical and physiological properties of the resultant gene products [3], [6]. To evaluate the true prevalence of these A-to-I RNA editing sites, we selected 109 putative editing sites, including 25 previously confirmed editing sites from a recently published database [24]. These 109 sites were purported to recode pre-mRNAs in one or more brain samples, and the frequencies of RNA editing ranged from 2% to 100% [24]. For our studies, three independent sets of normal human brain samples were used to quantify RNA editing: the first represents sample derived from the cerebral cortex and cerebellum pooled from 10 normal humans (Clontech); the second comprises one subject with 5 different brain regions sampled (Stanley Medical Research Institute; SMRI); and the third is of 5 normal humans with two brain regions sampled (Human Brain Collection Core, Center for Psychiatric Neuroscience (CPN), University of Mississippi Medical Center). As previously detailed [31], we combined multiple samples in the same sequencing lanes via barcoding to permit identification of individual samples (for details, see the materials and methods). DNA fragments containing the possible editing sites were amplified by reverse transcription–polymerase chain reaction (RT-PCR), and each amplicon was subjected to uHTS using an Illumina Genome Analyzer II platform (see Fig. 1A for the complete processing and analysis scheme). All of 109 amplicons mapped uniquely to the human genome, and none of them overlapped with pseudogenes which might confound the study as shown in previous reports [27], [32].

A total of 115 million reads were obtained from the three sets of human brain samples. Reads containing “Ns”, or short reads with matching sequence shorter than 30 bp, were filtered out. After two rounds of stringent filtering, 96.2 million reads were subsequently mapped to the target sequences. The average coverage for each site and each sample was 57,797±714 reads, and 95.2% editing sites from three sets of brain samples had greater than a 10,000 read coverage (Fig. 1B; for details of coverage for each site see Tables S1, S2, S3, S4). The A-to-G error rate for each editing site, which could be introduced either during PCR amplification or sequencing, was measured by quantifying the frequency of A-to-G misreads in unedited sites, and was estimated to be 0.08%±0.005%. Accordingly, an RNA editing frequency equal to or less than 0.08% is considered to be “background” in our system. A summary of the Illumina sequencing data is listed in Table 1.

**Table 1:** Summary of Illumina sequencing data.

Samples	Total reads	Mapped reads	Coverage^a^	A-to-G error rate^b^
2 normal human brain samples (Pooled, Clontech)	18,086,205	16,235,725	84,787±2,059	0.083%±0.011%
5 (SMRI) and 10 (CPN) normal human brain samples	96,677,697	79,992,527	54,300±710	0.074%±0.006%
72 psychiatric disorders and control human brainsamples (CPN)	141,226,208	91,482,049	53,458±939	0.070%±0.004%

a Coverage is the number of reads covered in each site, presented with Mean ± SEM.

b A-to-G error rate was measured by quantifying the frequency of A-to-G misreads in unedited sites, presented with Mean ± SEM.

Based on the consistency between the samples and the background A-to-G error rate in the system, three categories of editing sites were identified. Category I consists of sites with an editing frequency greater than 1%, which included 11 of the ‘new’ sites identified by Li et al [24] and 25 previously documented RNA editing sites (Fig. 1C, Table 2 and S1). Category II contains sites with an editing frequency between 0.08% and 1%, and included four sites identified by Li et al [24]: AEBP1, KCNQ5 KIF1A and PTPRN2 (Table S2). Category III is comprised of sites with an average editing frequency below our observed background error rate of 0.08%, which included 65 sites identified by Li et al [24] and is considered to be comprised of non-edited false positives (Table S3). Four sites (ATXN7, BIN1, C1ORF175 and RSU1), which we found to be single nucleotide polymorphisms (SNPs) rather than editing events, was excluded from the three categories (Table S4). To rule out the possibility of rare SNPs and mutations in the genome at the editing sites, which may confound *bona fide* RNA editing, we used PCR to amplify 15 sites from the matched mRNA and genomic DNA samples from the same individuals, and sequenced them by Sanger sequencing. In the matched samples from three individuals we examined, an unambiguous trace of guanosine was present at the editing sites with the editing frequency above 10% quantitated by our approach in RNA samples, whereas the genomic DNA showed only the presence of adenosine (Fig. S3).

**Figure 2:**
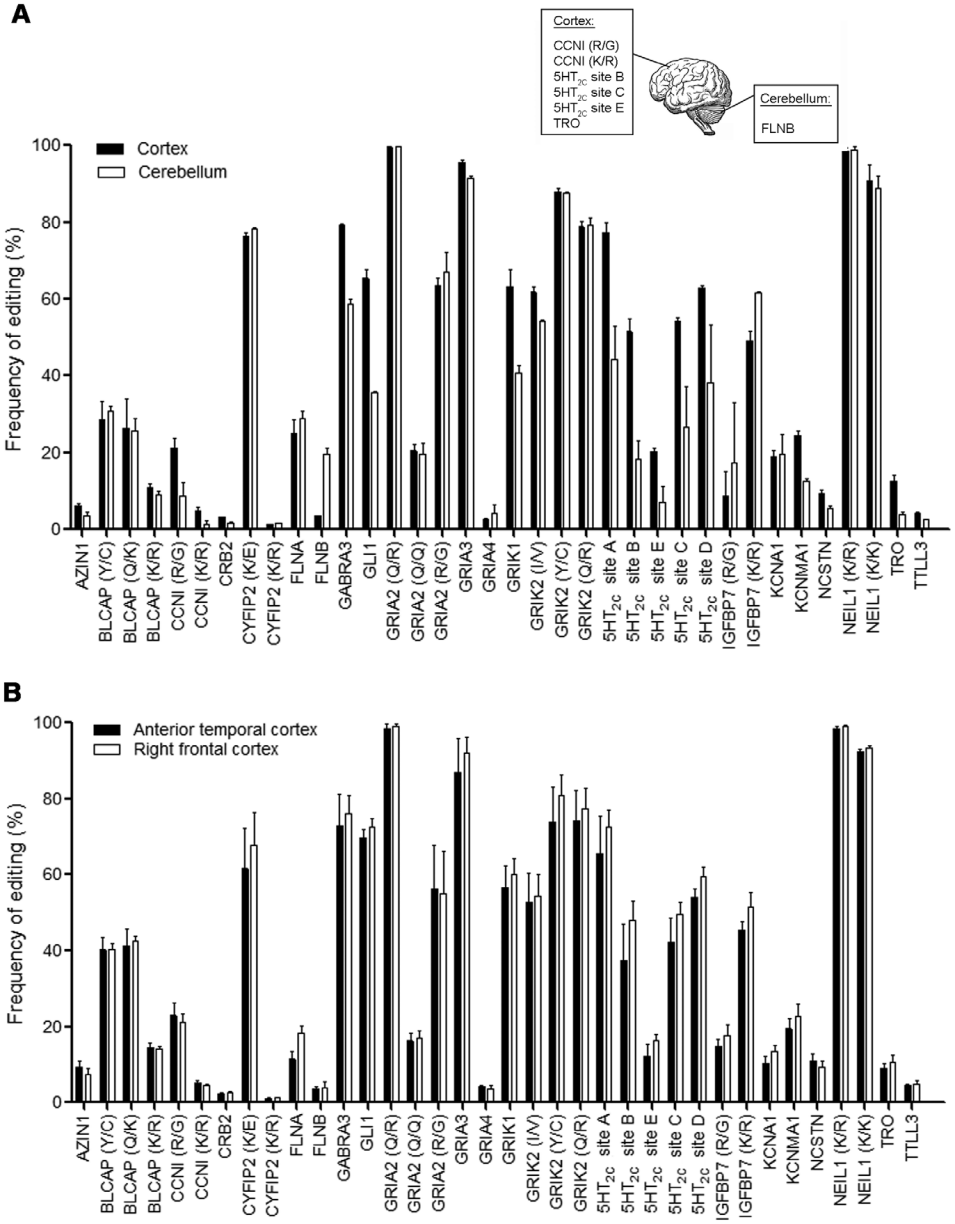
A-to-I RNA editing is different between the cortex and cerebellum, but not different between two regions of cortex, in human brain. **A**. Shows the distribution of A-to-I RNA editing frequency of 36 sites from category I, including 11 of the ‘new’ sites identified by Li et al (ref 24) and 25 previously ‘known’ sites, in the cortex and cerebellum from two sets of normal human brain samples. The RNA editing frequency is presented as a percentage of the total population of transcripts. A two-fold increase of RNA editing frequency at 6 sites (CCNI (R/G), CCNI (K/R), 5HT2C (siteB), 5HT2C (siteC), 5HT2C (siteE) and TRO) in cortex and one site (FLNB) in cerebellum are shown in the inset. **B**. Shows the distribution of A-to-I RNA editing frequency of 36 sites from category I, in the anterior temporal cortex (ATC) and the right frontal cortex (RFC) from the third set of normal human samples (n = 5). The RNA editing frequency is presented as a percentage of the total population of transcripts. No significant difference was found between these two regions of cortex.

Previous studies have shown differences in the frequency of RNA editing in different brain regions [33], [34], [35]. Accordingly, in the first and second sets of normal human brain samples, we re-analyzed the frequency of RNA editing of 36 sites from category I in the cerebral cortex and cerebellum. Due to the low sample number (n = 2), no statistics were done for this comparison; however, a general trend for decreased RNA editing was found in the cerebellum compared to the cortex. Notably, the frequencies of RNA editing at 7 sites, CCNI (R/G), CCNI (K/R), FLNB, 5HT2C (site B), 5HT2C (site C), 5HT2C (site E), and TRO, were more than two fold different between the cerebellum and the cortex (Fig. 2A and Table S1). In contrast, there was no significant difference in the frequency of RNA editing at 36 sites from category I between the anterior temporal cortex (ATC) and the right frontal cortex (RFC) from the third set of normal human samples (n = 5) (Fig. 2B). The difference in RNA editing between the cortex and cerebellum suggest that A-to-I RNA editing may play an important physiological role in different brain regions. However, due to the low sample number (n = 2) in present study, a larger number of samples will be needed to be examined to definitively address this issue.

Of 109 putative RNA editing sites [24], we were able to confirm 40 sites (36.7%), 36 sites from category I and 4 sites from category II, to be truly edited in the human brain samples we evaluated. These sites have been shown in the previous study to be edited in one or more brain samples, with frequencies of RNA editing ranged from 2% to 100% [24]. Several factors likely contribute to the high apparent false positive rate (63.3%) of A-to-I RNA editing in prior study. Firstly, in the prior report, the sequencing coverage for each site was quite low (36.1% sites had less than 10 reads, 73.2% sites had less than 100 reads and 95.2% sites had less than 1,000 reads in all tissue samples); indeed, some sites ***with as few as 10 total reads per tissue*** were considered to have adequate sequence coverage in their study [24]. In our study, by contrast, the coverage for each site was much higher than prior study, 95.2% RNA editing sites had more than 1×10^4^ reads and only 1.9% sites had less than 1,000 reads in three sets of brain samples. One general problem of all current next generation sequencing (NGS) reads is their higher error rate compared with Sanger sequencing [36]. It has been well described in the literature that the accuracy is dramatically diminished by low coverage in the NGS platform [37]. To accurately measure the frequency of RNA editing, which may range from 0% to 100%, a much higher coverage is required to compensate for sequencing error. Secondly, the relative fidelity of DNA amplification with *Taq* DNA polymerase (e.g., as used by Li et al. [24]) is low, and this will potentially significantly affect the results, as incorrect nucleotides can be incorporated during the initial amplification steps. In our study, the highest fidelity DNA polymerase available was used in the initial cDNA amplification, and its fidelity is at least 50-fold higher than that of *Taq* DNA polymerase (http://www.neb.com/nebecomm/products/productM0530.asp). Thirdly, some SNPs (single nucleotide polymorphisms) appear to be mis-annotated as RNA editing sites, *e.g.,* ATXN7 (GenBank ID: rs1053338), Bin1 (GenBank ID: rs138047593) (Table S4), C1ORF175 (GenBank ID: rs75269200) and RSU1 (GenBank ID: rs11539866). The A-to-G rate of ATXN7 is 13.21% in the cerebellum and 5.11% in the cortex from the pooled samples, and is ∼50% in two brain regions from same subject. The A-to-G rate of BIN1 is 7.58% in the pooled cerebellum sample and is around “background” level in other samples (Table S4). Thus, the highly inconsistent rates of A-to-G substitution between samples suggest that single nucleotide polymorphisms rather than RNA editing may be responsible. Based on our results, it appears that the A-to-I RNA editing frequencies of 109 putative sites reported in the previous study [24] may be overestimated in human brain. However, we can’t exclude the possibility that the discrepancy may be due to different samples used in our study and previous study.

### A-to-I RNA Editing in Schizophrenia, Major Depressive Disorder and Suicide

Previous studies have shown that the frequencies of RNA editing in GluR2 [38], [39], GRIK2 [40] and 5HT_2C_ receptor [41], [42], [43], [44], [45], [46] were altered in human psychiatric disorders. Alterations in RNA editing of the 5HT_2C_ receptor have been implicated in schizophrenia, major depressive disorder and in suicide. However, the results have been highly inconsistent among studies and, given the methodology used, inconclusive [41], [42], [43], [44], [47], [48] (for recent review see [49]). To address the potential significance of RNA editing in human psychiatric disorders, 29 editing sites from 19 genes (including the 5HT_2C_ receptor) were selected from Category I (*vide supra*) and examined in five groups of a total of 72 subjects: age-, sex-, tissue pH- and post-mortem interval (PMI)-matched controls (n = 15), major depressive disorder (MDD, n = 15), MDD with suicide (n = 15), schizophrenia (n = 15) and schizophrenia(7)/schizoaffective disorder(5) with suicide (n = 12). The right prefrontal cortex (Brodmann Areas 8/9) was sampled. There were no significant differences in age, gender, PMI and tissue pH among the groups (Table S5).

A total of 141 million reads were obtained from the 72 human samples, and of these, 91.5 million reads were mapped to the target sequences. The coverage for each site and each sample averaged 53,458±939 reads and the average A-to-G error rate was calculated to be 0.07% ±0.004% (Table 1). The editing frequency for each site from this cohort of 72 brain samples is thus consistent with the aforementioned three sets of normal brain samples. A general trend for decreased RNA editing was found in the MDD and schizophrenia groups compared to the control group, while no significant difference were identified at any sites (Fig. S1 and Table S6).

**Table 2:** A-to-I RNA editing sites with the frequency of editing above 1% in three sets of normal human samples.

Gene Name	Site	Genomic position^a^	Frequency of RNA editing^b^ (Mean±SEM)	Reads^c^ (Mean±SEM)
AZIN1^d^	S/G	chr8:103910812	6.96±0.88	71,192±6,463
BLCAP	Y/C	chr20:35580986	36.84±1.76	13,040±2,405
BLCAP	Q/K	chr20:35580977	37.22±2.43	13,040±2,406
BLCAP	K/R	chr20:35580947	12.96±0.68	13,040±2,407
CCNI^d^	R/G	chr4:78198704	19.73±1.71	92,647±6,897
CCNI^d^	K/R	chr4:78196188	4.25±0.40	99,327±5,241
CRB2^d^	T/A	chr9:125172441	2.63±0.24	36,336±3,848
CYFIP2	K/E	chr5:156669386	69.83±4.38	72,192±4,190
CYFIP2^d^	K/R	chr5:156669387	1.24±0.08	72,192±4,191
FLNA	Q/R	chrX:153233144	18.83±2.09	59,725±4,313
FLNB^d^	Q/R	chr3:58116841	5.48±1.44	58,461±4,517
GABRA3	I/M	chrX:151108975	74.08±3.20	65,175±5,507
GLI1^d^	R/G	chr12:56150891	64.89±3.14	51,897±4,292
GRIA2	Q/Q	chr4:158477325	99.09±0.43	81,668±4,010
GRIA2	Q/R	chr4:158477329	17.83±0.98	81,668±4,011
GRIA2	R/G	chr4:158500744	58.91±4.75	24,308±2,737
GRIA3	R/G	chrX:122426643	91.59±3.00	35,143±1,593
GRIA4	R/G	chr11:105309904	3.65±0.38	13,651±2,085
GRIK1	Q/R	chr21:29875621	56.92±2.67	72,065±3,592
GRIK2	I/V	chr6:102444382	55.78±2.92	52,960±3,377
GRIK2	Y/C	chr6:102444395	81.79±3.52	52,960±3,378
GRIK2	Q/R	chr6:102479281	77.26±2.89	42,994±3,236
5HT_2C_	A (I/V)	chrX:113988938	68.02±4.11	63,901±4,434
5HT_2C_	B(I/M)	chrX:113988940	41.78±4.15	63,901±4,435
5HT_2C_	E(N/D)	chrX:113988944	15.12±1.60	63,901±4,436
5HT_2C_	C(N/S)	chrX:113988945	45.87±3.16	63,901±4,437
5HT_2C_	D(I/V)	chrX:113988950	56.21±2.63	63,901±4,437
IGFBP7	R/G	chr4:57671043	14.90±2.52	1,236±919
IGFBP7	K/R	chr4:57670991	49.76±1.93	29,167±3,948
KCNA1	I/V	chr12:4892003	14.59±1.38	62,447±3,825
KCNMA1^d^	S/G	chr10:79067304	20.66±1.57	43,534±3,777
NCSTN^d^	S/G	chr1:158586611	9.08±0.90	75,689±6,413
NEIL1	K/R	chr15:73433139	98.80±0.25	7,651±1,655
NEIL1	K/K	chr15:73433140	92.12±0.88	7,651±1,656
TRO^d^	S/G	chrX:54972292	9.55±0.95	52,312±7,660
TTLL3^d^	K/R	chr3:9851560	4.17±0.39	54,057±4,098

a Genomic position is the position in human genomic database from UCSC (http://genome.ucsc.edu, hg18 version, March 2006 assembly).

b Frequency of RNA editing is presented as the percentage of the total population of transcripts.

c Reads is the number of transcripts sequenced.

d 11 new RNA editing sites identified by Li et al [24].

Although several previous studies [41], [42], [43], [46], [47] have reported alterations of editing of 5HT_2C_ serotonin receptor mRNA in both schizophrenia and MDD, our results demonstrated no significant differences between these groups in the brain samples we studied, although a slight decrease of editing at sites C, D and E occurred in the MDD and schizophrenia groups compared to controls (Table S6 and S7). When we separately analyzed the 24 protein isoforms of the 5HT_2C_ receptor produced by RNA editing, no significant difference were also identified between these groups at any isoforms of 5HT_2C_ serotonin receptor (Fig. S2 and Table S8).

**Figure 3:**
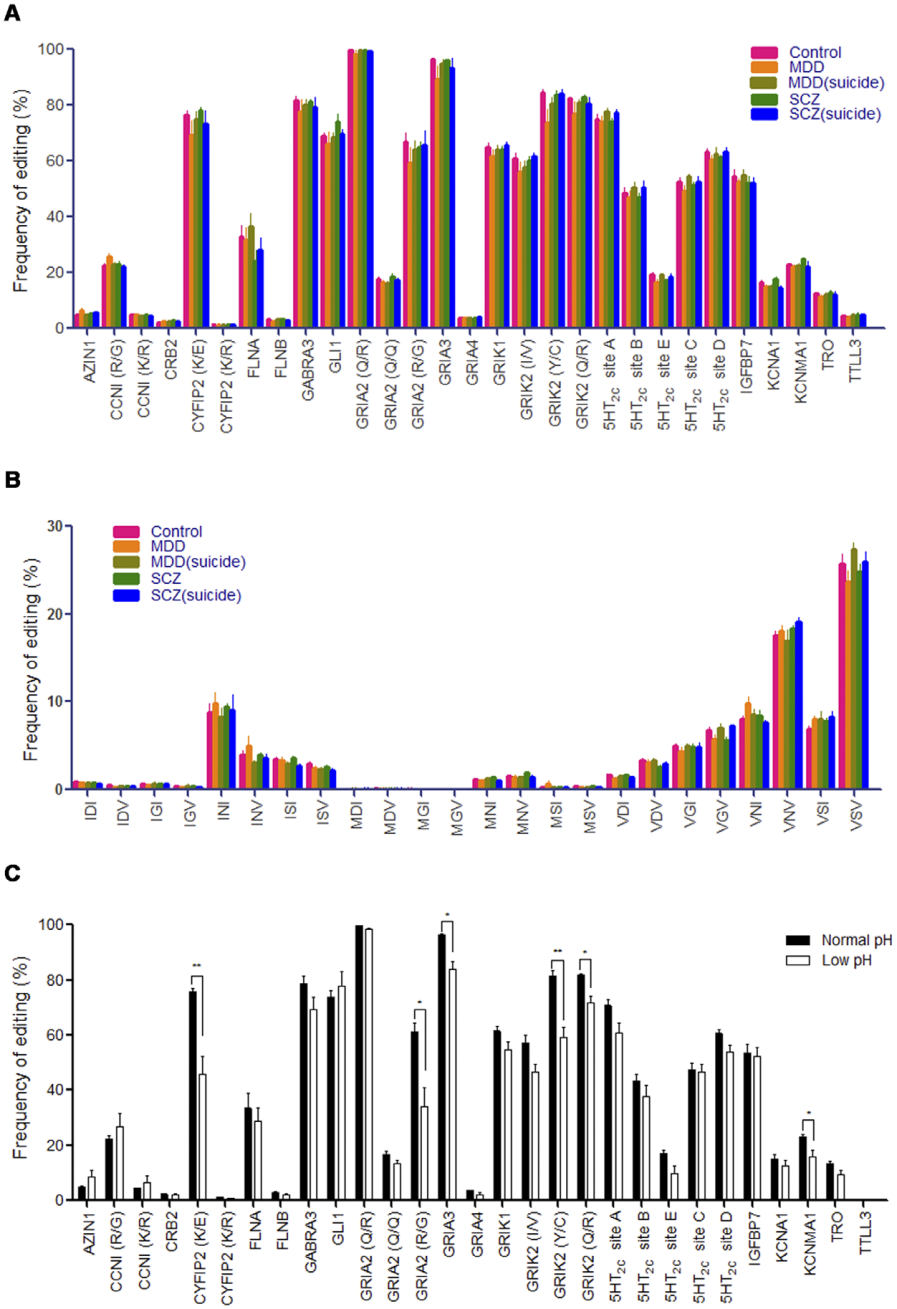
A-to-I RNA editing in brain is not altered in various psychiatric disorders. **A**. Shows A-to-I RNA editing frequency of 29 sites from category I in psychiatric patients and normal controls, excluding 8 samples with pH <6.1. RNA editing frequency is presented as mean, expressed as a percentage of the total population of transcripts, ± SEM. The data were analyzed by t-test with Benjamini–Hochberg correction for multiple comparisons using a P value of 0.05 as the criterion for statistical significance. The editing frequency in patients did not differ significantly from controls for any site tested. **B**. Shows the expression patterns of 24 isoforms of the 5HT_2C_ receptor produced by RNA editing in psychiatric patients and normal controls, excluding 8 samples with pH<6.1. The RNA editing frequency is presented as mean, expressed as a percentage of the total population of transcripts, ± SEM. The data were analyzed by s t-test with Benjamini–Hochberg correction for multiple comparisons using a P value of 0.05 as criterion of statistical significance. Editing frequency in patients did not differ significantly from controls for any site tested. **C**. Shows the frequency of A-to-I RNA editing of 29 sites from category I was examined in 8 samples with pH<6.1 and 8 matched samples with pH ≥6.1. The RNA editing frequency is presented as mean, expressed as a percentage of the total population of transcripts, ± SEM. The data were analyzed by t-test with Benjamini–Hochberg correction. Significant differences between the normal pH group and low pH group are shown by asterisks (*p<0.05; **p<0.01), and were seen in 6 of 29 sites examined.

RNA integrity is a major concern in studies using postmortem brain tissues and this may be affected by agonal factors, such as the specific agonal conditions at the time of death and agonal duration [50]. The pH of postmortem brain tissue has been reported to be inversely related to the agonal state at the time of death [51]. In our study, there is a slight, but insignificant, lower pH value in MDD and schizophrenia groups compared to other groups, which coincides with a trend for decreased RNA editing in these two groups. Harrison et al [51] have found that prolonged agonal states may produce brain pH values <6.0 and have recommended the exclusion of samples with a pH value <6.1. Thus, here we used a pH of 6.1 as cutoff to separate the subjects into two subgroups, low pH (<6.1) and normal pH (≥6.1). We found that 8 of 72 brain samples, distributed among all five subject groups, had low pH values. Those samples were then sub-grouped *post-hoc*, and eight matched samples with normal pH were selected as controls. A dramatic decrease of RNA editing was found in the low pH subgroup (*e.g.,* CYFIP (K/E) p<0.01, GRIA2 (R/G) p<0.05, GRIA3 p<0.05, GRIK2 (Y/C) p<0.01, GRIK2 (Q/R) p<0.05, KCNMA1 p<0.05) compared to the normal pH subgroup, and there was a statistically significant difference between the two subgroups (paired *t*-test, p<0.001) (Fig. 3C and Table S10). Therefore, the frequency of RNA editing observed in post-mortem samples appears to be inversely related to the tissue pH and agonal state. Prolonged low brain pH in the agonal state could conceivably inactivate ADARs and decrease the efficiency of RNA editing. The alterations of RNA editing of the 5HT_2C_ receptor observed in previous studies [41], [42], [43], [46], [47], thus may be due to differences in the agonal state of the subjects. As tissue pH affects the efficiency of RNA editing, 8 subjects with low pH (<6.1) were excluded from our study in a separate *post-hoc* analysis (Table S7). After reanalyzing the data, the conclusions regarding RNA editing of the 29 sites were not significantly changed, presumably due to the even distribution of low pH samples through all five groups. No significant differences were identified between these groups at all RNA editing sites we evaluated (Fig. 3A and Table S7) and any isoforms of 5HT_2C_ serotonin receptor in the brain samples we studied upon re-analysis of the data. (Fig. 3B and Table S9).

In summary, we have systematically and quantitatively examined 109 putative coding region A-to-I RNA editing sites by ultra-high-throughput sequencing (uHTS) in human brain. Only 40 of 109 sites identified by Li’s (ref 24) study were confirmed in our brain samples, which may suggest an overestimation of these RNA editing sites in the human brain. After analyzing the prefrontal cortex samples from MDD and schizophrenia in our study, we did not find any significant alterations in the frequency of RNA editing at any RNA editing sites we evaluated, including the sites of the 5HT_2C_ receptor transcripts, also in contrast with many previous studies. Our findings, however, do indicate that uHTS is a fast, powerful and quantitative method for assessing RNA editing in human tissues provided adequate attention is paid to experimental variables including tissue pH, estimations of sequencing and amplification errors, and statistical power considerations. uHTS of known RNA editing sites on multiple samples, as done in the present study, provides a more robust testing of focused hypotheses than more-or-less random whole-genome sequencing of one or even a few samples, with its attendant low coverage at each site. The results neither support many previous findings that large-scale alterations in RNA editing occur in psychiatric disorders nor the recent proposals that large numbers of genes contain coding regions is RNA edited.

## Materials and Methods

### Human RNA and Tissue Samples and Psychiatric Assessment

Human cortex and cerebellum total RNA from pooled normal human control samples (n = 10) were obtained from Clontech. Tissue samples from a normal control subject containing 5 different brain areas (premotor cortex (Brodmann areas 6), motor cortex (Brodmann area 4), parietal cortex (Brodmann area 7), occipital cortex (Brodmann area 19), and cerebellum) were obtained from the Stanley Medical Research Institute (SMRI, Chevy Chase, Maryland). The age, PMI, and average tissue pH of this subject was 37 years, 50 hours, and pH 6.61. Tissue samples from a normal control cohort containing 5 subjects, two brain regions (right anterior temporal cortex and right prefrontal cortex (Brodmann areas 38 and 8/9, respectively)) were obtained from the Human Brain Collection Core (Center for Psychiatric Neuroscience, University of Mississippi Medical Center, Jackson, Mississippi). The average age, PMI, and tissue pH of the cohort was 48.4±12.15 years, 20.49±6.63 hours, and pH 6.57±0.3. Samples from a 72 patient cohort (one brain region - right prefrontal cortex (Brodmann areas 8/9)) was obtained from the Human Brain Collection Core and consisted of five groups: normal controls (n = 15), major depressive disorder (MDD) (n = 15), major depressive disorder who committed suicide (MDD suicide) (n = 15), schizophrenia (SCZ) (n = 15), schizophrenia (7)/schizoaffective disorder (5) who committed suicide (SCZ suicide) (n = 12). Detailed information for this cohort is shown in Table S5.

All tissues from the Human Brain Collection Core were collected at autopsy at the Cuyahoga County Coroner’s Office, Cleveland, OH, using an ethical protocol approved by the Institutional Review Board of the University Hospitals of Cleveland and the University of Mississippi Medical Center. Informed written consent was obtained from the next-of-kin for all subjects. Informed consent for tissue donation at autopsy and a subsequent psychiatric assessment was sought from the legally-defined, adult next-of-kin of the deceased. The next-of-kin of the deceased were contacted by telephone, informed of the broad goals of the study and asked to participate by permitting sampling of tissue as well as agreeing to an interview held about three months later. If the family declines to participate, they are not re-contacted and no tissue is collected. If the family indicates a willingness to participate, written consent is obtained shortly thereafter by a research coordinator, after which the coroner releases the tissues for dissection, freezing and storage. Included with the informed consent form is a request to obtain medical records of the deceased. An appropriate, IRB-approved HIPAA Authorization Form is used in requesting relevant personal health information from doctors and/or hospitals identified by the next-of-kin or coroner’s records. If written consent is not received, no tissue is sampled nor are the families contacted again. All potential participants who do not consent to the study are not disadvantaged in any way by not participating in the study. After an initial contact, those who decline to participate are not contacted again. Cortical tissues were frozen in isopentane cooled by dry ice and stored at −80°C. The causes of death – natural, accidental or suicide – were determined by the coroner. Toxicology screening of postmortem blood and urine was performed by the coroner’s office. An antidepressant medication was present in four subjects with MDD and five subjects with schizophrenia. An antipsychotic medication was present in four subjects with schizophrenia. A trained interviewer assessed Axis I psychopathology for each subject by a structured clinical interview with knowledgeable next-of-kin, as previously described [52]. Consensus diagnosis was reached during meeting when all available information was reviewed by a psychiatrist, clinical psychologist, and social worker, and discussed until a consensus was reached. None of the control subjects had ever met criteria for an Axis I major mental illness. All 30 depressed subjects met diagnostic criteria for major depressive disorder (MDD) according to the Diagnostic and Statistical Manual of Mental Disorders IV (DSM-IV,4^th^ ed, 1994). All depressed subjects met criteria for a major depressive episode within the last two weeks of life except for three non-suicides: two depressed subjects were in partial remission and one was in full remission. Twenty two and five subjects met DSM-IV criteria for schizophrenia and schizoaffective disorder, respectively. Among depressed subjects committing suicide, the following comorbid diagnoses were noted: alcohol dependence (2), alcohol abuse (2), alcohol and cannabis abuse (1). Among depressed subjects not committing suicide, the following comorbid diagnoses were noted: opiate dependence (1), cannabis dependence (1), alcohol dependence and polysubstance abuse (1), polysubstance abuse (2). Among subjects with a psychotic disorder committing suicide, the following comorbid diagnoses were noted: cannabis dependence (1), polysubstance abuse (1) and alcohol dependence and cannabis abuse (1). Among subjects with a psychotic disorder not committing suicide, the following comorbid diagnoses were noted: alcohol dependence and cannabis abuse (2) alcohol dependence (1) and cannabis abuse (1).

### RNA Extraction and RT-PCR

Trizol (Invitrogen) was used to extract RNA from ∼123 mg of each human brain sample. Ten µg of RNA was treated with DNAse (DNA-free, Ambion), and 2 µg of the DNase-treated RNA was added to a reverse transcription reaction performed using the SuperscriptTM III RNase H Reverse Transcriptase kit (Invitrogen) with random hexamer primers (Invitrogen). The cDNA product was used as template to generate a double stranded DNA fragment by PCR for use in high throughput sequencing experiments.

One µL of cDNA (of 20 µL) was used as template for a 20 µL PCR reaction (conditions: 1 cycle −98°C, 1 min; 35 cycles −98°C 10 sec, 68°C 15 sec, 72°C 15 sec; 1 cycle −72°C 5 min) to amplify a fragment containing the edited region of interest using Phusion Hot Start High-Fidelity DNA Polymerase (New England BioLabs). The error rate of Phusion DNA Polymerase is 4.4×10^−7^ (http://www.finnzymes.fi/pcr/phusion_products.html). PCR fragments (106–233 base pairs in length) were diluted 6X, and 5 µL were used as template for a second round of PCR (50 µL reaction) (the conditions for this 2^nd^ round were the same as for the 1^st^ round). PCR fragments were purified using the QIAquick96 PCR Purification kit (Qiagen) and 5 ul of product was run on a 1.5% agarose gel to determine quality and quantity of amplification. In order to confirm amplification of the correct gene with the correct tag, 5 ul of each PCR product was submitted for Sanger sequencing. The primers used for amplification are listed in Table S11. Each primer used for amplification contained adapter sequences necessary for cluster generation. In addition, the forward primer contained a sequence corresponding to a sequence primer optimized by Illumina for use in the Genome Analyzer II (uppercase letters not in bold), as well as a sample-identification “barcode”. All “barcodes” used in the experiment were listed in Table S12, which were designed with 3 A/Ts and 3 G/Cs.

### Ultra High Throughput Sequencing

For sequencing of the Clontech samples, two “barcodes” were used (cortex and cerebellum); PCR products of 96 genes from two samples were mixed in equal parts and designated for one lane in the Genome Analyzer II flow cell. Five “barcodes” were used for the five-patient, two-brain area normal control cohort samples from the Human Brain Collection Core, as well as the one-patient, five brain-area normal control samples from the Stanley Medical Research Institute (SMRI) to differentiate a similar number of genes and editing sites as above for each tissue sample. PCR products from five samples were then mixed in equal parts creating three mixed sample sets, each set designated for one lane in the Genome Analyzer II flow cell. For the cohort of the 72 human psychiatric and control samples, 12 different “barcodes” were used to differentiate 19 genes and 29 edited sites for each sample, which were then mixed in equal parts to create 6 sample sets, each set designated for one lane in the Genome Analyzer II flow cell. The process of Ultra high throughput sequencing was similar to our previous publication. Briefly, purified PCR products were diluted to a concentration of 15 nM. 2 µL of the diluted PCR product was used for denaturation (total volume 20 µL). 4 µL of the denaturation mixture was diluted in 996 µL of hybridization solution. The hybridization mixture (final DNA concentration about 6 pM) was loaded into the Cluster Station for cluster generation. Primer hybridization was performed on the Cluster Station using 6.6 µL of 500 nM sequencing primer (Primer sequence: 5′-acactctttccctacacgacgctcttccgatct-3′) diluted in 1313 µL of hybridization buffer. Cluster generation was performed for 76 cycles followed by base-by-base sequencing initiated by the sequencing primer on the Genome Analyzer II. The Genome Analyzer II uses two different lasers to excite the dye attached to each nucleotide. Since the emission spectra of these four dyes overlap, the four images thus obtained are not independent. As in Sanger sequencing, the frequency cross-talk is deconvolved using a frequency cross-talk matrix. Therefore, the crosstalk matrix calculation requires control lanes for samples with skewed base compositions. Thus, a control human genomic DNA sample was run in parallel on the same flowcell concurrently with the human RNA editing samples.

### Data Analysis and Statistics

Sequences were analyzed using a Perl 5 script (available for download at http://pdsp-temp.pha-med.unc.edu/Download/code.php) written by us to filter the data through two rounds of filters and sort the data that passed through the filters. Reads containing “Ns”, or short reads mapped to the target region shorter than 30 bp, were filtered out. The mapped reads equal or large than 30 bp were counted. The percentage of RNA editing was calculated by the number of reads containing “G” at the editing site divided by the total number of reads containing “A” or “G” at the editing site. The human genomic reference sequence for analysis was downloaded from UCSC (http://genome.ucsc.edu, hg18 version, March 2006 assembly). The A-to-G error rate for each editing site investigated was determined by measuring the rate of A-to-G misreads within a few base pairs of each editing site. Further data analysis was performed in Microsoft Excel and Graphpad Prism 5.0. All statistical analyses were performed in Graphpad Prism 5.0. For purposes of making statistical comparisons in the cohort of 72 human psychiatric and control samples, all reads generated from one sample were treated as one experiment (N = 12−15 for each sub cohort). The data were analyzed by two-tailed *t*-test and subsequent Benjamini–Hochberg correction for multiple comparisons using a P value of 0.05 as criterion of statistical significance.

## Supporting Information

Figure S1 **A-to-I RNA editing frequency of 29 sites from category I in psychiatric patients and normal controls, including 8 samples with pH <6.1.** RNA editing frequency is presented as mean, expressed as a percentage of the total population of transcripts, ± SEM. The data were analyzed by t-test with Benjamini–Hochberg correction for multiple comparisons using a P value of 0.05 as criterion of statistical significance.
(TIF)

Figure S2 **Expression pattern of 24 isoforms of the 5HT_2C_ receptor produced by RNA editing in psychiatric patients and normal controls, including 8 samples with pH<6.1.** The RNA editing frequency is presented as mean, expressed as a percentage of the total population of transcripts, ± SEM. The data were analyzed by t-test with Benjamini–Hochberg correction for multiple comparisons using a P value of 0.05 as criterion of statistical significance.
(TIF)

Figure S3 **Validation of RNA editing sites by Sanger sequencing.** 15 RNA editing sites were verified by Sanger sequencing with the matched genomic DNA and cDNA samples from the same individuals. RNA editing was indicated by a trace of guanosine in cDNA sequence, while the genomic DNA sequence shown only adenosine signals. The RNA editing sites with the frequency above 10% measured by our approach have shown a clear signal of guanosine.
(TIF)

Table S1 **Category I RNA editing sites with >1% A-to-I RNA editing in three sets of normal human samples.** RNA editing frequency is presented as the percentage of the total population of transcripts. Three independent sets of normal human samples were used in this study. The first is the cortex and cerebellum pooled from 10 normal humans; the second comprises one subject (ID: S343) with 5 different brain regions sampled (Stanley Medical Research Institute; SMRI) and the third is of 5 normal humans (ID:228-451, 228-612, 228-695, 229116, 244-079) and two brain regions, anterior temporal cortex (ATC) and right frontal cortex (RFC, Brodmann areas 8&9) (Human Brain Collection Core). 11 new RNA editing sites are shown in red. The values of RNA editing frequency in the cerebellum, which were more than 1.5 fold different from the cortex, were highlighted.
(XLSX)

Table S2 **Category II RNA editing sites with 0.08%–1% A-to-I RNA editing in three sets of normal human samples.** RNA editing frequency is presented as the percentage of the total population of transcripts. Three independent sets of normal human samples were used in this study. The firstt is the cortex and cerebellum pooled from 10 normal humans; the second comprises one subject (ID: S343) with 5 different brain regions sampled (Stanley Medical Research Institute; SMRI) and the third is of 5 normal humans (ID:228-451, 228-612, 228-695, 229116, 244-079) and two brain regions, anterior temporal cortex (ATC) and right frontal cortex (RFC, Brodmann areas 8&9) (Human Brain Collection Core). The values of RNA eidting frequency above 0.08% were highlighted.
(XLSX)

Table S3 **RNA editing frequency (%) in category III sites with <0.08% A-to-I RNA editing in three sets of normal human samples.** RNA editing frequency is presented as the percentage of the total population of transcripts. Three independent sets of normal human samples were used in this study. The first is the cortex and cerebellum pooled from 10 normal humans; the second comprises one subject (ID: S343) with 5 different brain regions sampled (Stanley Medical Research Institute; SMRI) and the third is of 5 normal humans (ID:228-451, 228-612, 228-695, 229116, 244-079) and two brain regions, anterior temporal cortex (ATC) and right frontal cortex (RFC, Brodmann areas 8&9) (Human Brain Collection Core).
(XLSX)

Table S4 **Four single nucleotide polymorphisms (SNP) were misannotated as RNA editing in three cohorts of normal human samples.** RNA editing frequency is presented as the percentage of the total population of transcripts. Three independent sets of normal human samples were used in this study. The first is the cortex and cerebellum pooled from 10 normal humans; the second comprises one subject (ID: S343) with 5 different brain regions sampled (Stanley Medical Research Institute; SMRI) and the third is of 5 normal humans (ID:228-451, 228-612, 228-695, 229116, 244-079) and two brain regions, anterior temporal cortex (ATC) and right frontal cortex (RFC, Brodmann areas 8&9) (Human Brain Collection Core). The values of A-to-G rate greater than 0.08% were highlighted.
(XLSX)

Table S5 **Characterization of 72 human psychiatric and control samples.** The values are presented as mean ± SEM. PMI: post-mortem interval.
(XLSX)

Table S6 **A-to-I RNA editing frequency (%) of 29 editing sites from 19 genes in normal and psychiatric disease patients.** RNA editing frequency is presented as mean, expressed as a percentage of the total population of transcripts, ± SEM. Data were analyzed by t-test with Benjamini–Hochberg correction for multiple comparisons using a P value of 0.05 as criterion of statistical significance.
(XLSX)

Table S7 **A-to-I RNA editing frequency (%) of 29 editing sites from 19 genes in normal and psychiatric diseases patients excluding 8 low pH samples.** RNA editing frequency is presented as mean, expressed as a percentage of the total population of transcripts, ± SEM. The data were analyzed by t-test with Benjamini–Hochberg correction for multiple comparisons using a P value of 0.05 as criterion of statistical significance.
(XLSX)

Table S8 **Distribution of the frequency (%) of 24 isoforms of 5HT2C receptors produced by RNA editing in normal and psychiatric disorder patients.** 5-HT2C receptor isoform frequency is presented as mean, expressed as a percentage of the total population of transcripts, ± SEM. The data were analyzed t-test with Benjamini–Hochberg correction for multiple comparisons using a P value of 0.05 as criterion of statistical significance.
(XLSX)

Table S9 **Distribution of the frequency (%) of 24 isoforms of 5HT2C receptors produced by RNA editing in normal and psychiatric disorder patients (excluding 8 low pH samples).** 5-HT2C receptor isoform frequency is presented as mean, expressed as a percentage of the total population of transcripts, ± SEM. The data were analyzed by t-test with Benjamini–Hochberg correction for multiple comparisons using a P value of 0.05 as criterion of statistical significance.
(XLSX)

Table S10 **A-to-I RNA editing frequency (%) of 29 editing sites from 19 genes in normal pH (pH >6.1) and low pH (pH <6.1) samples.** RNA editing frequency is presented as mean, expressed as a percentage of the total population of transcripts, ± SEM. Data were analyzed by t-test with Benjamini–Hochberg correction for multiple comparisons using a P value of 0.05 as criterion of statistical significance. The significant differences between the control group and other groups are shown by asterisks (*p<0.05; **p<0.01).
(XLSX)

Table S11 **The primers used in the study.** All primers are in the 5′-to-3′ direction from left to right. One “barcode” (CAGCTA) was used to illustrate this set of primers. See Table S11 for all “barcodes” used in the study.
(XLSX)

Table S12 **The “barcodes” used in the study.**
(XLSX)
