# Supplementary figures and images for: Quantitative Analysis of Focused A-To-I RNA Editing Sites by Ultra-High-Throughput Sequencing in Psychiatric Disorders
Source: PLoS One. 2012 Aug 17;7(8):e43227. doi: 10.1371/journal.pone.0043227 (PMC3422315; doi:10.1371/journal.pone.0043227)

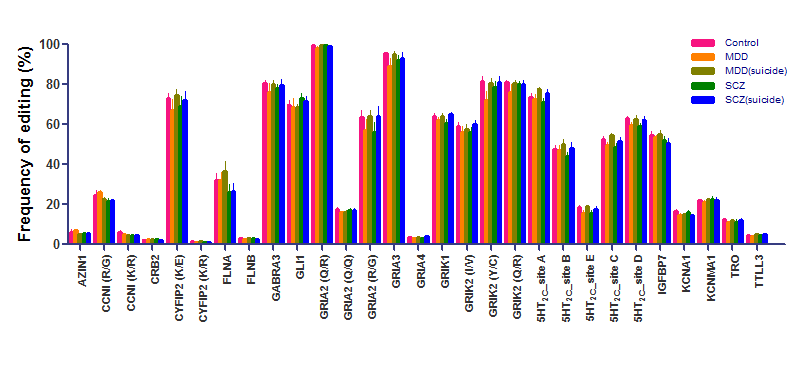

Supplement: Figure S1 — A-to-I RNA editing frequency of 29 sites from category I in psychiatric patients and normal controls, including 8 samples with pH <6.1. RNA editing frequency is presented as mean, expressed as a percentage of the total population of transcripts, ± SEM. The data were analyzed by t-test with Benjamini–Hochberg correction for multiple comparisons using a P value of 0.05 as criterion of statistical significance. (TIF) [file pone.0043227.s001.tif]

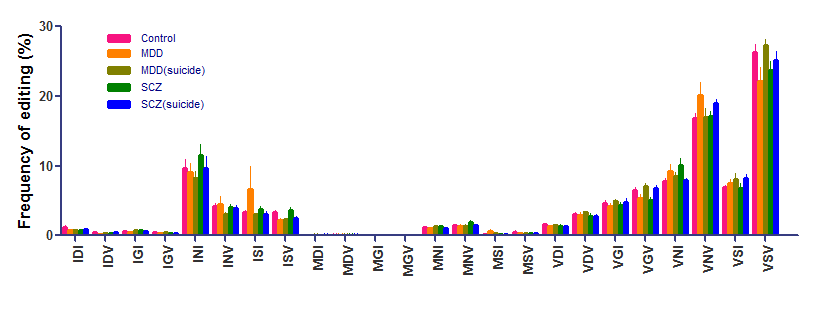

Supplement: Figure S2 — Expression pattern of 24 isoforms of the 5HT2C receptor produced by RNA editing in psychiatric patients and normal controls, including 8 samples with pH<6.1. The RNA editing frequency is presented as mean, expressed as a percentage of the total population of transcripts, ± SEM. The data were analyzed by t-test with Benjamini–Hochberg correction for multiple comparisons using a P value of 0.05 as criterion of statistical significance. (TIF) [file pone.0043227.s002.tif]

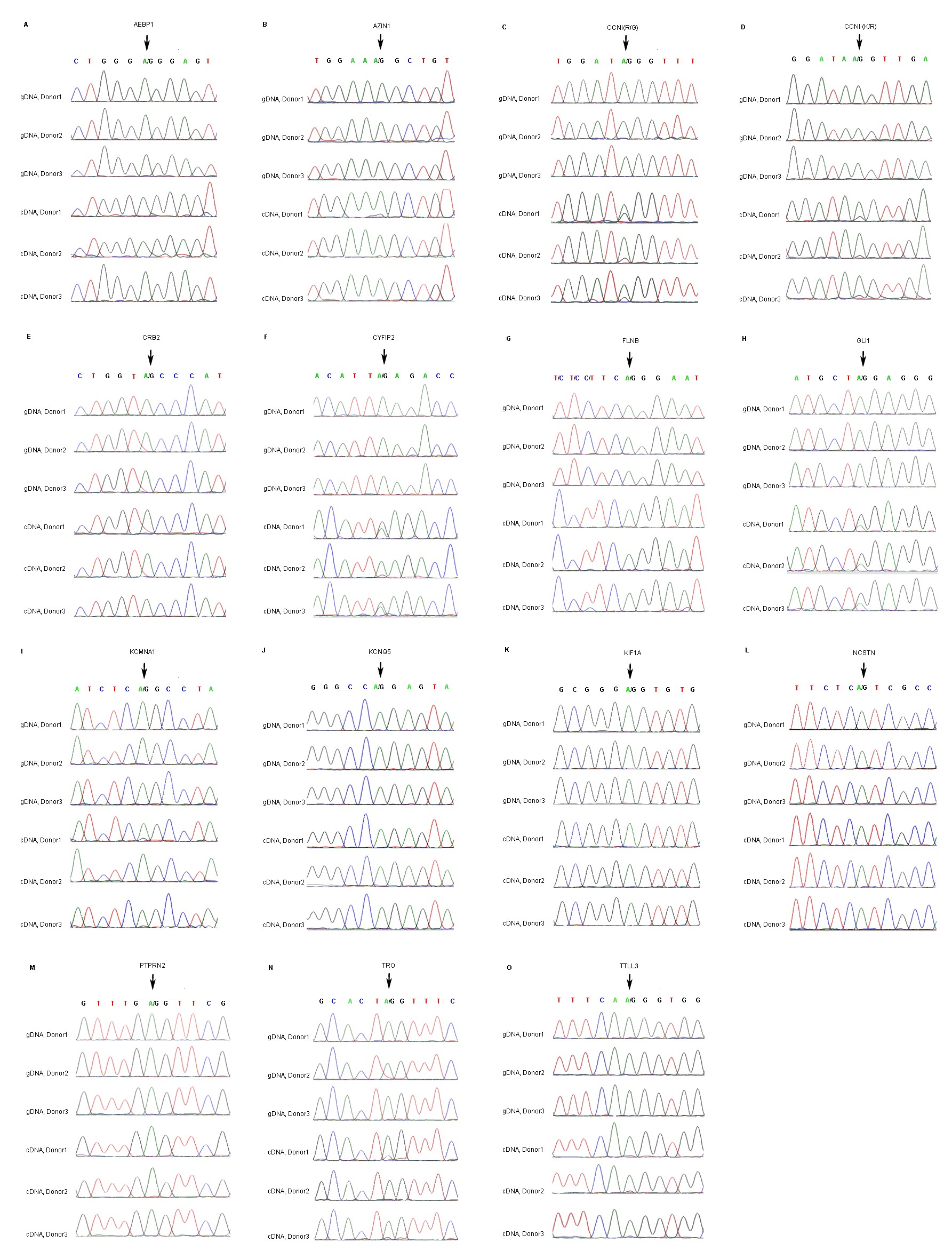

Supplement: Figure S3 — Validation of RNA editing sites by Sanger sequencing. 15 RNA editing sites were verified by Sanger sequencing with the matched genomic DNA and cDNA samples from the same individuals. RNA editing was indicated by a trace of guanosine in cDNA sequence, while the genomic DNA sequence shown only adenosine signals. The RNA editing sites with the frequency above 10% measured by our approach have shown a clear signal of guanosine. (TIF) [file pone.0043227.s003.tif]
